# Supplementary material for: The RNA interactome of human telomerase RNA reveals a coding-independent role for a histone mRNA in telomere homeostasis
Source: eLife. 2018 Oct 25;7:e40037. doi: 10.7554/eLife.40037 (PMC6249008; doi:10.7554/eLife.40037)
Supplement: Figure 1—source data 2. [file elife-40037-fig1-data2.docx]

| Gene | Name | Predicted interaction(s) | Inter-action site(s) in hTR (nt) | Interaction site(s) in target (GRCh38) | Notes |
| --- | --- | --- | --- | --- | --- |
| FLNA | Filamin A | CG  5’-GCCU GCCUGCCGCCUU-3’  \|\|\|\| \|\|\|\|\|\|\|\|\|\|\|\|  3’-CGGA CGGACGGCGGAA-5’  AA | 130-147 | ChrX(-)  154.354.986-154.355.012  (CDS) | hTR-target interaction verified by qRT-PCR (Figure 1 – figure supplement 2) |
| HIST1H1C | Histone cluster 1 H1 family member C | 5’-GGCUUCUCCGGAGGC-3’  \|\|\|\|\|\|:\|\|\|\|\|\|\|\|  3’-CCGAAGGGGCCUCCG-5’ | 270-284 | Chr6(-)  26.056.081-26.056.095  (CDS) | hTR-target interaction verified by qRT-PCR (Figure 3) |
| SCARNA2 | Small Cajal body-specific RNA2 |  |  |  | hTR-target interaction verified by qRT-PCR (Figure 9); refer to Figure 9 for the predicted interactions |
| RPL10 | Ribosomal protein L10 | C CAC  5’-GCCC GGGGCUU UCCGGAGGCACC UGC-3’  \|\|\|\| \|\|\|\|\|\|\| \|\|\|::\|\|\|\|\|\|\| :\|\|  3’-CGGG CCCCGAA AGGUUUCCGUGG GCG-5’  A A | 264-293 | ChrX(+)  154.400.483-154.400.506  (CDS) |  |
| EIF4G1 | Eukaryotic translation initiation factor 4 gamma 1 | 5’-UGGGAGGGGUGG-3’  :\|:\|\|\|\|:\|\|\|\|  3’-GCUCUCCUCACC-5’ | 20-31 | Chr3(+)  184.321.534-184.321.545  (CDS) |  |
| TPT1 | Tumor protein, translationally-controlled 1 | U C U C C A  5’-UG CAGCCG GGGU C CUCGGGGG GAGGG G GG-3’  \|\| \|\|\|\|\|\| \|:\|: \| \|\|\|\|\|\|\|\| \|\|\|\|\| \| \|\|  3’-AC GUCGGC CUCG G GAGCCCCC CUCCC C CC-5’  CC C  AG C CG  5’-UGUC CUGCUGGCC GUU CCCCUCC-3’  \|\|\|\| \|\|:\|\|\|:\|\| :\|: \|\|\|\|\|\|\|  3’-ACAG GAUGACUGG UAG GGGGAGG-5’  AA | 314-348  177-204 | Chr13(-)  45.341.138-45.341.169  (5’ UTR)  45.340.170-45.340.184 … 45.340.712-45.340.721  (CDS) | Two potential hTR binding sites are present in TPT1; the second binding site is interrupted by an intron; hTR-target interaction verified by qRT-PCR (Figure 1 – figure supplement 2) |
| AKAP2 | A-kinase anchoring protein | G U  5’-GCGGAGGG UGG CC UGGGA GGGGUGG GG-3’  \|\|\|\|\|\|\|\| \|\|\| \|\| \|\|\|\|\| \|\|\|\|\|:\| \|\|  3’-CGCCUCCC ACC GG ACCCU CCCCAUC CC-5’  U A U ACA U | 6-34 | Chr9(+)  110.136.287-110.136.320  (CDS) |  |
| AP2M1 | Adaptor related protein complex 2 mu 1 subunit | UU AGGG U  5’-GGG GCGG UGGGCCUG GGAGGGG GGUGGC-3’  \|\|\| \|\|\|\| \|\|\|:\|\|\|\| \|\|\|\|\|:\| \|\|\|\|\|\|  3’-CCC CGCC ACCUGGAC CCUCCUC CCACCG-5’  U GUGG A | 1-35 | Chr3(+)  184.183.647-184.183.680  (3’ UTR) |  |
| TTC3 | Tetratricopeptide repeat domain 3 | CCU  5’-GGGUGGG GGGAGG GGUGGUGGCCAUU-3’  \|\|\|\|\|\|\| :\|\|\|\|: \|\|\|\|\|\|\|\|\|\|\|\|\|  3’-CCCACCC UCCUCU CCACCACCGGUAA-5’  CU UA | 11-39 | Chr21(+)  37.202.469-37.202.498  (3’ UTR) |  |
| ARID1B | AT-rich interaction domain 1B | CC GA  5’-GGG UGG GGGGUGGUGGCC-3’  \|\|\| \|\|\| \|\|\|\|\|\|\|\|\|\|\|\|  3’-CCC ACC CCCCACCACCGG-5’  CC AA | 15-36 | Chr6(+)  157.201.375-157.201.396  (CDS) |  |
| TRAM2 | Translocation associated membrane protein 2 | A  5’-GGGUUGCGG GGGUGGGCCU-3’  :\|\|\|\|:\|\|\| ::\|\|\|\|\|\|\|\|  3’-UCCAAUGCC UUCACCCGGA-5’  G | 1-20 | Chr6(-)  52.498.473-52.498.494  (3’ UTR) |  |
| HSP90AB1 | Heat shock protein 90 alpha family class B member 1 | A C  5’-UGCGG GGGUGGG CUGGG-3’  \|\|\|\|\| \|:\|\|\|\|\| \|\|\|\|\|  3’-ACGCC CUCACCC GACCC-5’  AAC A | 5-23 | Chr6(+)  44.253.331-44.253.351  (CDS) | HSP90AB1-hTR interaction was reported by LIGR-seq (Sharma et al., 2016) |

**Figure 1 – source data 2**

**Details of the predicted RNA-RNA interactions for the transcripts listed in Figure 1B.**
